# Depression and comorbid obstructive sleep apnea: Association between positive airway pressure adherence, occurrence of self-harm events, healthcare resource utilization, and costs

**DOI:** 10.1016/j.jad.2023.12.055

**Published:** 2023-12-29

**Authors:** E.M. Wickwire, K.V. Cole, R.B. Dexter, A. Malhotra, P.A. Cistulli, K.L. Sterling, J.L. Pépin, medXcloud group

**Affiliations:** a Sleep Disorders Center, Division of Pulmonary and Critical Care Medicine, Department of Medicine, Department of Psychiatry, University of Maryland School of Medicine, Baltimore, MD, USA; b ResMed Science Center, San Diego, CA, USA; c ResMed Science Center, Halifax, Nova Scotia, Canada; d University of California San Diego, La Jolla, CA, USA; e Charles Perkins Centre, Faculty of Medicine and Health, University of Sydney, Department of Respiratory and Sleep Medicine, Royal North Shore Hospital, Sydney, Australia; f Univ. Grenoble Alpes, Institut National de la Santé et de la Recherche Médicale (INSERM) U 1300, HP2 Laboratory (Hypoxia: Pathophysiology), Grenoble Alpes University, Grenoble, France

**Keywords:** Depression, Obstructive sleep apnea, Healthcare resource use, Healthcare costs, Positive airway pressure therapy, Administrative claims

## Abstract

**Objective:**

Previous studies have shown that treatment of obstructive sleep apnea (OSA) with positive airway pressure (PAP) therapy in patients with depression may improve depression symptoms and response to antidepressant therapy. We investigated the association between PAP therapy adherence, self-harm events, healthcare resource utilization (HCRU), and costs over 2 years in a national sample of patients with pre-existing depression and newly diagnosed comorbid OSA.

**Methods:**

Administrative claims data were linked to objective PAP therapy usage. Inverse probability treatment weighting was used to compare outcomes over 2 years across PAP adherence levels. The predicted numbers of emergency room (ER) visits and hospitalizations by adherence level were assessed using risk-adjusted generalized linear models.

**Results:**

37,459 patients were included. Relative to non-adherent patients, consistently adherent patients had fewer self-harm events (0.04 vs 0.05, *p* < 0.001) after 1 year, and significantly (all p < 0.001) fewer ER visits (0.66 vs 0.86) and all-cause hospitalizations (0.13 vs 0.17), and lower total ($11,847 vs $11,955), inpatient hospitalization ($1634 vs $2274), and ER visit ($760 vs $1006) costs per patient in the second year of PAP therapy. Consistently adherent patients showed lower risk for hospitalizations and ER visits.

**Limitations:**

Using observational claims data, we were unable to assess clinical characteristics including sleep, sleepiness, and daytime symptoms, or important social determinants of health. We were limited in assessing care received outside of the included health plans.

**Conclusion:**

Consistent adherence to PAP therapy over 2 years was associated with improved HCRU outcomes for patients with pre-existing depression newly diagnosed with comorbid OSA.

## 1. Introduction

By any standard, depression incurs a massive disease burden in the U.S. and worldwide (Leopold, 2003; Liu et al., 2020; Stewart et al., 2003). Identifying factors that can reduce the burden of depression is an important research priority with many gaps in knowledge. One modifiable factor that could potentially reduce the burden of depression, if treated, is obstructive sleep apnea (OSA). OSA is a common and costly chronic medical condition that impacts approximately 27 % of men and 9 % of women in the United States and approximately one billion people worldwide (Benjafield et al., 2019; Peppard et al., 2013). This sleep-related breathing disorder is characterized by repetitive breathing pauses throughout the night, each lasting a minimum of ten seconds, with associated blood oxygen desaturations, increased sympathetic activation, and cortical arousals during sleep. Untreated OSA is associated with a broad range of adverse health consequences, including increased risk for cardiovascular disease (Konecny et al., 2010; Mehra et al., 2006; Peppard et al., 2000) stroke (Chan et al., 2010; Yaggi et al., 2005), metabolic syndromes (including Type 2 diabetes) (Drager et al., 2010; Punjabi et al., 2004; Reichmuth et al., 2005), and premature death (Cowie et al., 2021; Marshall et al., 2014; Mehra et al., 2022; Punjabi et al., 2009; Redline et al., 2023; Yeghiazarians et al., 2021), as well as dramatically increased economic burden including health care resource utilization (HCRU) and costs (American Academy of Sleep, 2016; Wickwire et al., 2019; Wickwire et al., 2020). Importantly, OSA is also associated with diminished health-related quality of life and worsened mental health, including depression.

Disturbed sleep is one of the nine core symptoms of major depressive disorder (*American Psychological Association*, 2019), and OSA is a well-known comorbidity of depression and other mood disorders. Prior research has suggested a bidirectional relationship between OSA and depression, and multiple studies have demonstrated elevated rates of OSA among patients with depression with pooled prevalence estimates of comorbid OSA among patients with depression ranging from 36.3 % to 48 % (Gupta and Simpson, 2015; Haddad and Chen, 2017; Sharafkhaneh et al., 2005; Stubbs et al., 2016; Wickwire and Albrecht, 2023). More important, comorbid OSA is associated with worsened depressive outcomes, including risk for suicide and self-harm (intentional damage or injury to one’s body) (Udholm et al., 2022), as well as poorer response to depression treatment. For example, in a study of patients undergoing medication therapy for depression and relative to patients without OSA, participants with comorbid OSA were 37 % less likely to respond to treatment (i.e., 44 % of patients without OSA responded, but only 28 % of patients with comorbid OSA responded) (Waterman et al., 2016). Likewise, another study of medication therapy for depression found that relative to participants without OSA, individuals with comorbid OSA remained significantly more depressed after ten weeks (Roest et al., 2012).

In addition, OSA has been associated with worsened econommic outcomes among older adults with depression (Wickwire and Albrecht, manuscript accepted for publication).

Evidence suggests that treatment of OSA can improve depressive symptoms and improve response to depression treatment. For example, among patients evaluated for OSA, depressive symptoms are common and improve following OSA treatment with positive airway pressure (PAP) therapy, the most prescribed treatment for OSA (Edwards et al., 2015; Walker et al., 2021). Similarly, data from randomized clinical trials (RCTs) demonstrate a beneficial effect of PAP therapy on depression outcomes. In a secondary analysis of data from the RICCADSA (Randomized Intervention with CPAP in Coronary Artery Disease and Sleep Apnea) trial, Balcan and colleagues found that CPAP reduced depressive symptoms among patients with depression, non-sleepy OSA, and cardiovascular disease, with gains maintained at 12 months (Balcan et al., 2019). More recently, Jackson and colleagues performed a RCT among individuals with clinically diagnosed depression and comorbid OSA (Jackson et al., 2021). OSA treatment with PAP therapy was associated with reduced severity of depression symptoms, reduced rate of meeting diagnostic criteria for depression, and reduced antidepressant medication usage (Jackson et al., 2021). These beneficial effects are especially noteworthy given that depression is well-known to reduce medication adherence across a range of conditions and has been associated with reduced adherence with PAP therapy in some but not all studies (Wickwire et al., 2013).

Despite these findings, little is known about the impact of PAP therapy on population-level health and economic outcomes among individuals with depression and comorbid OSA. To address this important gap in knowledge, the purpose of the present study was to determine the relationship between PAP adherence and subsequent occurrence of self-harm events, HCRU, and costs among a national sample of individuals with depression and newly diagnosed comorbid OSA. Our primary hypothesis was that PAP adherence is associated with reduced self-harm, HCRU, and costs.

## 2. Methods

### 2.1. Data source

This was a retrospective cohort study conducted among patients with depression and newly diagnosed with OSA between September 2014 and April 2019. De-identified payer-sourced (“closed”) administrative claims data containing >100 geographically dispersed health plans across the United States (licensed from Inovalon Insights LLC, Bowie MD) were linked with objective PAP usage data (AirView^™^; ResMed Corp, San Diego, CA). The databases were linked through a tokenization process, and the resulting linked database underwent a third-party expert determination to ensure compliance with the Health Insurance Portability and Accountability Act. The study design was reviewed by an Institutional review Board (Advarra, Ref number Pro0004005) and deemed to be exempt from oversight.

### 2.2. Study cohort

The study cohort consisted of adults (age ≥ 18 years) who completed a sleep test and subsequently received an International Classification of Diseases-10 (ICD-10) OSA diagnosis within 60 days. Patients were required to have at least 1 year of claims data prior to the first sleep test and 2 years of claims data after setup with an AirSense10^™^ PAP device (ResMed Corp, San Diego, CA). Depression was identified by the presence of at least two healthcare encounter claims with an ICD-10 diagnosis of depression or at least one inpatient hospitalization with a diagnosis of depression in the year prior to device setup. Exclusion criteria included evidence of PAP supplies in the year prior to the sleep test; or any of the following during the study period: a diagnosis of central sleep apnea, nocturnal hypoventilation, pregnancy, or end-stage renal disease; or dialysis use. Operational definitions are listed in Supplemental Tables 1 and 2.

### 2.3. PAP adherence

PAP usage was objectively measured by the PAP device for each night over two years. The US Centers for Medicare and Medicaid Services (CMS) considers a patient compliant with therapy if the PAP device is used at least 4 h per night on 70 % of nights during a consecutive 30-day period within the first 90 days of therapy. Three levels of adherence were defined: 1) adherent patients who met CMS criteria for all eight consecutive 90-day timeframes (quarters) within the first two years; 2) non-adherent patients who did not meet CMS criteria in any of the eight quarters; and 3) intermediate adherent patients who met CMS criteria in at least one but no more than seven quarters.

### 2.4. Outcomes

HCRU was defined as counts of claims across multiple points of service: office visits, emergency room (ER) visits, all-cause hospitalizations, depression-related hospitalizations, 30-day all-cause hospital readmissions, depression-related specialist visits, and self-harm events. Healthcare costs were categorized into those related to inpatient hospitalizations, outpatient hospitalizations, ER visits, and total costs. Proxy costs for all resource use were provided by Inovalon Insights LLC based on their proprietary Proxy Financials algorithm, which is based on CMS Medicare prospective payment system fee schedules. Depression-related hospitalizations were defined as a hospitalization that included a diagnosis of depression in the primary position on the claim. 30-day all-cause readmissions were defined as hospitalizations that occur within 30 days of a previous hospitalization. Specialist visits were defined as office visits related to depression-based specialists, including psychotherapy visits and health and behavior visits. Self-harm events were defined based on the claims-based definition provided by Udholm and colleagues (Udholm et al., 2022). Codes used to identify these visits in the administrative claims data are listed in Supplemental Table 3.

### 2.5. Covariates

The following variables were used to control for baseline differences between PAP adherence groups: 1) demographics (age, gender, payer); 2) obesity based on BMI and obesity ICD-10 codes; 3) comorbidities based on ICD-10 diagnosis codes (Supplemental Table 1) in the year prior to the first sleep test (coronary artery disease, atrial fibrillation, atrial flutter, other arrhythmia, hypertension, pulmonary hypertension, cerebrovascular disease, heart failure, asthma, chronic obstructive pulmonary disease, pneumonia, psychotic disorders (bipolar and non-bipolar), anxiety, other mood disorders, insomnia, type 2 diabetes, hyperlipidemia, gastroesophageal reflux disease, cancer, fibromyalgia, chronic fatigue syndrome); 4) adherence to antidepressant medication; and 5) prior year all-cause hospitalizations and ER visits.

Adherence to antidepressant medications was used as a proxy to control for the effects of healthy behaviors, using a similar approach to previous research studies that have evaluated adherence to statin medications (Platt et al., 2010). In the current study, pharmacy claims were used to identify prescription fills of antidepressants in accordance with current guidelines (American Psychological Association, 2019), including selective serotonin reuptake inhibitors (SSRIs), serotonin and norepinephrine reuptake inhibitors (SNRIs), tricyclic antidepressants, monoamine oxidase inhibitors (MAOIs), and atypical antidepressants. Among patients who filled ≥1 antidepressant prescription 181–360 days prior to PAP initiation, those with a proportion of days covered (PDC) ≥80 % were considered “adherent to antidepressants”, while those with a PDC < 80 % were considered “not adherent to antidepressants”. Those without any fills were assumed to not have been prescribed antidepressants.

### 2.6. Analytical plan

First, based on baseline covariates, a multinomial logistic regression model was used to generate propensity scores estimating the likelihood of being in each adherence group were calculated. Using these scores, inverse probability of treatment weighting (IPTW) was applied to balance adherence groups on baseline covariates to help control for confounding (Austin and Stuart, 2015; Zhou et al., 2020). The IPTW approach weights the sample so that the distribution of covariates is similar across adherence groups and mirrors the distribution of the overall cohort. Balance between groups was assessed using standardized mean differences, with a value <0.1 indicating good balance (Austin and Stuart, 2015). To assess the association between adherence level and outcomes, pairwise differences (adherent vs intermediate, adherent vs non-adherent, intermediate vs non-adherent) in the mean number of healthcare visits and mean healthcare costs were assessed using weighted Wilcoxon rank-sum tests.

Next, to evaluate the impact of PAP adherence on two-year all-cause hospitalizations and two-year ER visits, negative binomial regression models were developed (zero-inflated models were used when supported by the data). Initially, all demographics and covariates were considered for these models. Final model covariates were selected based on statistical significance for a given outcome, taking into account considerations such as Akaike information criteria and multicollinearity. Model goodness of fit was assessed by McFadden’s grouped Log-likelihood R-squared (LL-R^2^) and 90th percentile predicted range. From these models, the number needed to treat (NNT) was calculated as 1/absolute risk reduction. NNT represents the number of patients that would need to improve their level of adherence to PAP therapy to avoid one additional event, i.e., non-adherent to intermediate, intermediate to adherent, or non-adherent to adherent.

All analyses were performed using R statistical software version 4.0.3, PSWeight R package (R-CoreTeam, 2021; Zhou et al., 2020).

## 3. Results

### 3.1. Baseline characteristics

The final sample included 37,459 patients with depression and newly diagnosed comorbid OSA. Of these, 62 % were female and the average age was 50.9 (±12.2) years (Table 1). The most common comorbidities at baseline were hypertension (experienced by 62.0 % of patients), hyperlipidemia (55.6 %), anxiety (55.9 %), gastroesophageal reflux disease (39.3 %), type 2 diabetes (28.6 %), and insomnia (28.0 %). The majority (68.7 %) of patients were prescribed one or more antidepressant medications. In these patients, 67.8 % were prescribed SSRIs, 43.7 % atypical antidepressants, 32.1 % SNRIs, 10.6 % tricyclic antidepressants, and 0.1 % MAOIs. In terms of HCRU, 99.7 % had at least one office visit, 46.6 % had a specialist visit, 43.8 % had an ER visit, 15.4 % had an all-cause hospitalization, 3.6 % had a self-harm event, 1.9 % had a 30-day hospital readmission, and 1.2 % had a depression-related hospitalization in the year prior to initiating PAP therapy.

### 3.2. PAP adherence

During the first two years following PAP initiation, 27.1 % of patients (*n* = 10,172) were considered adherent to PAP therapy, 44.0 % (*n* = 16,471) were intermediately adherent, and 28.9 % (n = 10,816) were non-adherent. In the entire cohort, 64.5 % met the CMS compliance criteria in the first 90 days. Mean PAP usage for each adherence group is listed in Table 2. PAP usage was consistently higher in the adherent group in the first and second year. Those intermediately adherent to PAP therapy over 2 years have lower adherence over time – starting at 85 % adherent in Q1, then reducing to 22 % adherent in Q8.

As depicted in Table 1, baseline characteristics were well balanced between adherence levels after weighting, with all standardized mean differences between adherent/intermediate, adherent/non-adherent, and intermediate/non-adherent patients presenting <0.1.

### 3.3. Healthy user effect: adherence to antidepressant medications

For patients taking antidepressants, 72.0 % were categorized as adherent to their medication and 28.0 % were not adherent. 77.2 % of PAP adherent patients were also adherent to antidepressants versus 72.4 % of intermediate patients and 66.2 % of non-adherent patients. Thus, adherence to antidepressants was included as a covariate in all models and was well-balanced with the IPTW adjustment.

### 3.4. Association between PAP adherence and occurrence of self-harm events, healthcare resource utilization, and costs

After applying IPTW, relative to non-adherence, PAP adherence was associated with reduced occurrence of self-harm events (0.04 vs 0.05, *p <* 0.001) over one year. The trend remained (0.04 vs 0.05) but diminished in significance at two years (*p* = 0.14; Table 3).

**Fig. 1.**
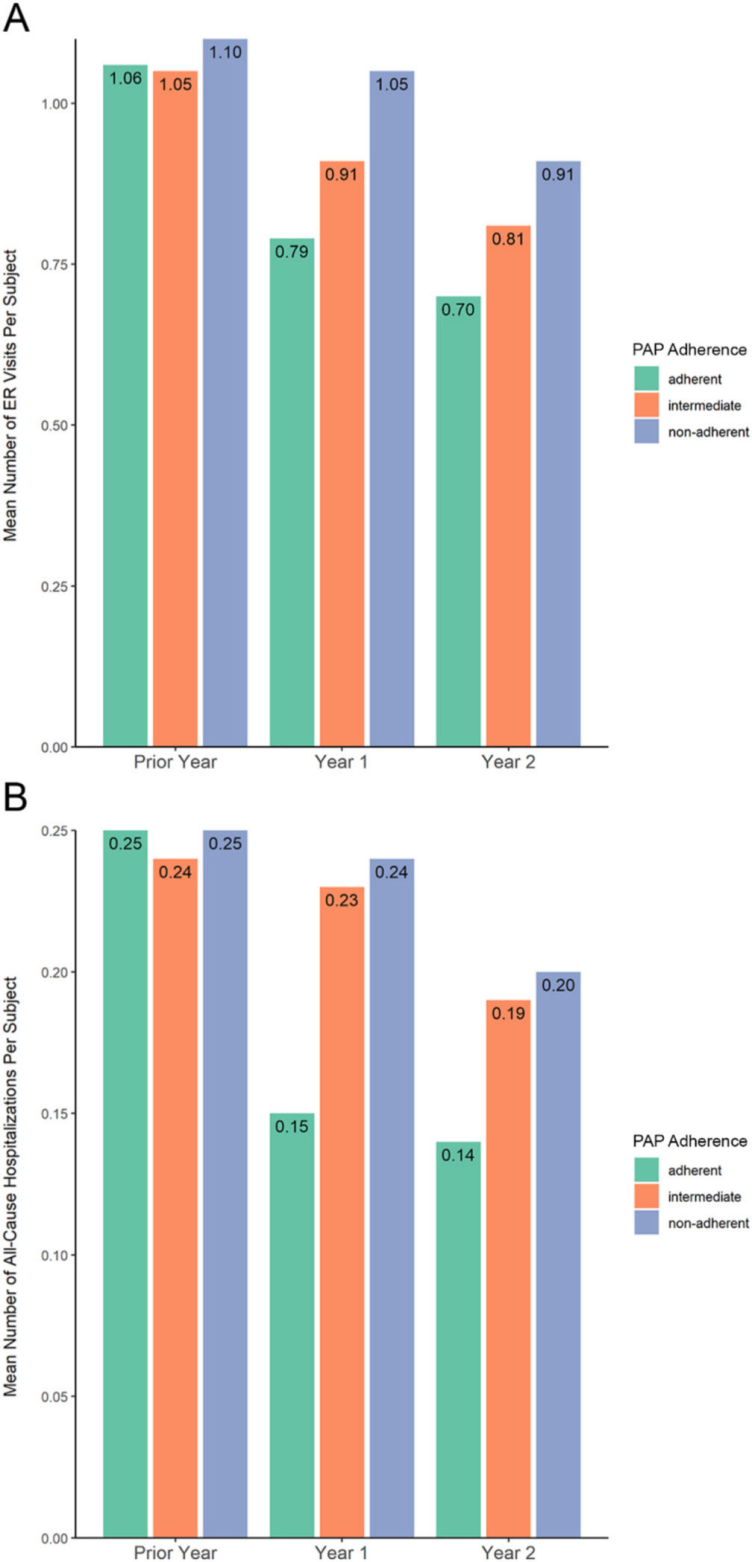
Healthcare Resource Use in Year Prior to, 1^st^, and 2^nd^ Years of PAP Therapy with Inverse Probability Treatment Weighting. (A) ER Visits. (B) All-Cause Hospitalizations.

In terms of HCRU, relative to non-adherent patients and intermediate patients, adherent patients had significantly fewer ER visits, all-cause hospitalizations, and 30-day hospital readmissions in year 2 (Fig. 1, Table 3). When comparing intermediate to non-adherent patients, results are similar between the two groups with notably fewer ER visits in the intermediate group at 1 and 2 years.

Total healthcare costs, inpatient hospitalizations, and ER visit costs were all significantly lower in year 2 for adherent patients compared to non-adherent patients (Table 3). Outpatient hospitalization costs were not significantly different for any adherence comparison in year 2. ER visit costs significantly decreased with increasing PAP adherence in both years 1 and 2.

### 3.5. Risk-adjusted outcomes

The risk-adjusted model for mean number of hospitalizations over 2 years fit reasonably well (LL-R^2^ = 0.064) and was left uncapped. Converting 6.3 non-adherent or 12.4 intermediate patients to being adherent would avoid one hospitalization. The risk-adjusted model for mean number of ER visits over 2 years also fit reasonably well (LL-R^2^ = 0.105) and was capped at the 95th percentile. Converting 0.9 non-adherent or 1.9 intermediate patients to being adherent would avoid one ER visit. Adherent patients had a lower risk of a hospitalization or ER visit through 2 years of PAP therapy compared to intermediate and non-adherent patients.

## 4. Discussion

This national study is the first to examine the impact of PAP adherence on population-level health and economic outcomes among adults with depression and comorbid OSA. Our most important clinical finding is that relative to non-adherence, consistent PAP adherers was associated with a 20 % reduction in self-harm events at one year (with similar trends at two years). In terms of economic outcomes, two years following PAP initiation, objective PAP adherence was associated with reduced HCRU and costs across multiple points of service. In terms of economic outcomes, relative to non-adherers high adherers demonstrated 23 % fewer ER visits and 24 % fewer hospitalizations as well as lower total, inpatient, and ER costs ($11,847 vs $11,955; $1634 vs $2274; $760 vs $1006, respectively) at two years. Indeed, even partial (intermediate) adherence was associated with reduced HCRU and costs, suggesting potential economic benefit from even lower PAP usage among individuals with depression. Taken together, these findings offer clinical implications and suggest several directions for future research.

Results from the present study build upon and expand past findings that PAP therapy is associated with improved depressive outcomes among individuals with depression and comorbid OSA (Balcan et al., 2019; Edwards et al., 2015; Jackson et al., 2021; Walker et al., 2021). Results from several systematic reviews and meta-analyses of observational studies have found that PAP therapy reduces depression symptoms (Gupta et al., 2016; Zheng et al., 2019). Further, these results have been confirmed in RCTs (Jackson et al., 2021). For example, in a meta-analysis of RCTs (Yang et al., 2020), relative to participants in control conditions, individuals receiving PAP demonstrate reduced depressive symptomatology. A sub analysis of the Sleep Apnea Cardiovascular Endpoints (SAVE) trial showed that PAP reduces depression in minimally symptomatic OSA with established cardiovascular diseases, and improvements in depression occurred independent of improvements in sleepiness. (Zheng et al., 2019) In the present study, a particularly noteworthy result is the association between PAP adherence and reduced occurrence of self-harm events, the most severe possible outcome of depression. Although the number of self-harm events in our study was relatively small, this finding is consistent with two recent Danish cohort studies, which found untreated OSA to be associated with an increased risk of self-harm and death by suicide, and PAP to be associated with reduced risks for these outcomes (Sweetman and Adams, 2022; Udholm et al., 2022). It is interesting to speculate potential mechanisms that might underlie the beneficial effects of PAP therapy. Broadly speaking, adherence to PAP therapy is associated with reductions in daytime sleepiness and domains of cognition, which might contribute to improved depressive outcomes observed in this study.

At the same time, although the present results highlight the potential benefit from successful treatment of comorbid OSA among individuals with depression, it is important to note that the U.S. Preventative Task Force has found insufficient evidence to support routine screening for OSA in asymptomatic individuals (Feltner et al., 2022). In this vein, the clinical practice guideline from the American Psychiatric Association is particularly relevant. This guideline specifically highlights OSA as a key comorbidity among patients with depression, particularly individuals who experience daytime sleepiness, fatigue, or treatment-resistant symptoms (Davidson, 2010). Thus to build on present results, future research should examine the clinical and economic impact of OSA screening, triage, and treatment on depression outcomes as well as mental health more broadly (Wickwire et al., 2020). Given the public health imperative to reduce the burden of depression, such insight could support data-driven decision making by health leaders and policy makers seeking to manage population health in the future.

In addition to our depression-specific findings, these data add to the growing body of evidence demonstrating a beneficial effect of treatment of comorbid OSA more broadly. For example, a 2019 systematic review found 15 of 17 comparisons to be associated with positive economic benefit from treatment of OSA, including comorbid OSA (Wickwire et al., 2019). Similarly, recent studies have found positive economic benefit associated with PAP adherence in patients with chronic obstructive pulmonary disease (Sterling et al., 2022) or Type 2 diabetes mellitus (Sterling et al., 2023), as well as reduced inpatient HCRU (Wickwire et al., 2022) and reduced 30-day readmissions (Bailey et al., 2022) among older adults with cardiovascular disease and comorbid OSA. Another recent paper reported a dose-response relationship between adherence to PAP therapies and healthcare utilization in OSA (Malhotra et al., 2023). In that study, economic benefits were seen even with PAP usage as low as 1–2 h/night. These findings are generally consistent with results from the present study, which demonstrate significant reductions in ER visits even in the intermediately adherent group.

Our study possesses several strengths. First, our integrated data source included both de-identified administrative claims as well as linked objective PAP usage. Second, our sample was large and included patients from all regions of the U.S., providing adequate statistical power and increasing generalizability of our findings. Third, in addition to a 12-month baseline period, we included two years of follow-up post PAP initiation to increase capture of outcomes of interest. Finally, we controlled for many potential covariates and employed a novel approach to the healthy user effect, using adherence to antidepressant medications as a proxy for healthy patient behavior.

At the same time, our study was limited in several aspects. First, although powerful, our linked administrative claims and objective PAP usage data source does not include measures of sleep, daytime symptoms, quality of life, patient motivations, or other clinical variables of interest. Second, access to mental health care is limited in the U.S., and we were unable to assess care received outside of the included health plans, such as pastoral counseling, self-pay therapies, or other approaches. Third, patients were required to possess continuing enrollment, thereby introducing a potential “survivor bias” in terms of self-harm and suicide-related outcomes. Fourth, we were unable to assess social determinants of health known to impact both depression and OSA care, including race, socioeconomic status, area socioeconomic deprivation, and other factors. Fifth, our analyses did not include Medicare fee-for-service patients, limiting our generalizability to this population. Finally, in using an observational study design, we were unable to make definitive conclusions about causality.

In conclusion, results from this national analysis found PAP adherence to be associated with improved health and economic outcomes including occurrence of self-harm events, HCRU, and costs among adults with depression and comorbid OSA. Future research should examine the health and economic impact of screening, triage, and treatment of OSA among individuals with depression.

## Supplementary Material

sup

## Figures and Tables

**Table 1:** Characterization by Positive Airway Pressure (PAP) Adherence from Year Prior to Starting PAP Therapy.

Characteristic	Unmatched Cohort				IPTW Matched Cohort		
	Overall	Adherent	Intermediate	Non-Adherent	Adherent	Intermediate	Non-Adherent
N	37,459	10,172	16,471	6803	10,172	16,471	10,816
Female	61.9 %	56.6 %	64.6 %	62.9 %	61.8 %	61.9 %	61.8 %
Age	50.9 (12.2)	52.3 (11.6)	51.0 (12.2)	49.6 (12.8)	51.5 (11.7)	50.9 (12.2)	50.4 (12.7)
Age groups							
18–54	58.9 %	54.2 %	58.8 %	63.3 %	58.9 %	58.9 %	59.1 %
55–69	35.4 %	39.6 %	35.5 %	31.1 %	35.4 %	35.3 %	35.3 %
≥70 Payer	5.8 %	6.2 %	5.7 %	5.5 %	5.7 %	5.8 %	5.7 %
Payer							
Commercial	62.1 %	75.0 %	62.8 %	49.0 %	61.9 %	62.1 %	62.1 %
Medicaid	25.3 %	13.6 %	24.3 %	37.7 %	25.9 %	25.3 %	25.6 %
Medicare Advantage	12.6 %	11.4 %	12.9 %	13.3 %	12.2 %	12.6 %	12.3 %
Obesity							
Morbidly Obese	34.7 %	32.1 %	34.8 %	37.1 %	34.7 %	34.7 %	34.9 %
Obese	29.3 %	30.7 %	29.2 %	27.9 %	29.3 %	29.3 %	29.3 %
Overweight	6.2 %	5.3 %	6.6 %	6.4 %	6.3 %	6.2 %	6.1 %
Not Categorized	29.9 %	31.9 %	29.4 %	28.6 %	29.6 %	29.9 %	29.6 %
Cardiac comorbidities							
Atrial Fibrillation	5.2 %	5.9 %	5.0 %	4.9 %	5.3 %	5.2 %	5.3 %
Atrial Flutter	1.0 %	1.2 %	1.0 %	0.8 %	1.1 %	1.0 %	1.1 %
Cerebrovascular Disease	7.4 %	6.3 %	7.2 %	8.9 %	7.2 %	7.5 %	7.5 %
Coronary Artery Disease	14.3 %	12.4 %	14.4 %	16.0 %	14.8 %	14.4 %	14.6 %
Heart Failure	7.6 %	6.1 %	7.5 %	9.3 %	8.0 %	7.7 %	7.8 %
Hypertension	62.0 %	59.9 %	61.6 %	64.5 %	62.2 %	62.0 %	62.2 %
Other Arrhythmia	8.4 %	8.2 %	8.2 %	8.9 %	8.6 %	8.4 %	8.5 %
Pulmonary Hypertension	2.5 %	2.2 %	2.5 %	2.8 %	2.7 %	2.5 %	2.6 %
Respiratory comorbidities							
Asthma	23.1 %	18.9 %	23.5 %	26.5 %	23.4 %	23.1 %	23.2 %
COPD	14.6 %	10.3 %	14.2 %	19.2 %	15.0 %	14.6 %	14.8 %
Pneumonia	6.4 %	5.4 %	6.5 %	7.2 %	6.4 %	6.4 %	6.5 %
Affective comorbidities							
Anxiety	55.9 %	50.5 %	56.5 %	60.2 %	55.9 %	55.9 %	56.0 %
Bipolar Disorder	9.2 %	5.4 %	9.5 %	12.4 %	9.6 %	9.3 %	9.3 %
Insomnia	28.0 %	22.8 %	28.8 %	31.7 %	27.9 %	28.1 %	28.2 %
Other Mood Disorders^a^	20.6 %	17.9 %	20.6 %	23.1 %	21.1 %	20.6 %	21.0 %
Psychotic Disorders	3.9 %	2.1 %	3.7 %	5.7 %	3.9 %	3.9 %	3.9 %
Other comorbidities							
Cancer	7.2 %	7.7 %	7.4 %	6.5 %	7.2 %	7.2 %	7.2 %
Chronic Fatigue Syndrome	6.2 %	5.6 %	6.7 %	6.0 %	6.2 %	6.2 %	6.3 %
Fibromyalgia	9.4 %	6.8 %	10.1 %	10.7 %	9.7 %	9.4 %	9.4 %
GERD	39.3 %	34.5 %	40.2 %	42.4 %	40.0 %	39.3 %	39.4 %
Hyperlipidemia	55.6 %	57.0 %	55.3 %	54.7 %	55.8 %	55.6 %	55.5 %
Type 2 Diabetes	28.6 %	25.1 %	28.2 %	32.7 %	29.1 %	28.7 %	28.8 %
Antidepressant medications							
Adherent	49.5 %	53.7 %	50.0 %	44.5 %	48.9 %	49.4 %	49.2 %
Not Adherent	19.2 %	15.8 %	19.0 %	22.7 %	19.4 %	19.2 %	19.4 %
Not Prescribed	31.3 %	30.5 %	30.9 %	32.8 %	31.7 %	31.4 %	31.4 %
Healthcare visits							
Office Visits	15.67 (13.58)	13.06 (11.59)	15.89 (13.66)	17.79 (14.76)	15.52 (13.88)	15.76 (13.72)	15.92 (13.59)
Specialist Visits	5.24 (11.35)	5.13 (11.57)	5.43 (11.70)	5.06 (10.55)	5.65 (11.99)	5.34 (11.66)	4.91 (10.57)
ER Visits	1.06 (2.48)	0.63 (1.45)	0.99 (2.13)	1.56 (3.47)	1.04 (2.09)	1.04 (2.26)	1.09 (2.74)
All-Cause Hospitalizations	0.23 (0.72)	0.18 (0.59)	0.22 (0.72)	0.29 (0.82)	0.23 (0.70)	0.23 (0.72)	0.24 (0.73)
30-Day Readmissions	0.03 (0.34)	0.02 (0.25)	0.03 (0.35)	0.04 (0.39)	0.03 (0.33)	0.03 (0.34)	0.03 (0.33)
Depression Hospitalization	0.02 (0.19)	0.01 (0.14)	0.02 (0.19)	0.02 (0.22)	0.02 (0.18)	0.02 (0.19)	0.02 (0.19)
Self-Harm Events	0.06 (0.40)	0.04 (0.37)	0.05 (0.37)	0.07 (0.48)	0.07 (0.66)	0.06 (0.39)	0.06 (0.42)
Healthcare costs							
Total	14,520 (23,271)	12,499 (18,450)	14,667 (27,846)	16,197 (19,183)	14,455 (19,466)	14,724 (27,791)	14,543 (17,575)
Inpatient Hospitalizations	2964 (10,487)	2429 (8990)	2882 (10,553)	3591 (11,603)	2964 (9819)	2986 (10,759)	3022 (10,556)
Outpatient Hospitalizations	2559 (4874)	2253 (4619)	2708 (5133)	2621 (4688)	2525 (4731)	2673 (5090)	2514 (4695)
ER Visits	1089 (2696)	691 (1894)	1042 (2489)	1533 (3466)	1071 (2424)	1084 (2606)	1105 (2820)

COPD, chronic obstructive pulmonary disease; ER, emergency room; GERD, gastroesophageal reflux disease; IPTW, inverse probability treatment weighted. PAP adherent patients met US Centers for Medicare and Medicaid Services (CMS) criteria for eight of eight quarters over two years; intermediate patients met CMS criteria in at least one but no more than seven quarters; non-adherent patients did not meet CMS criteria for any of the eight quarters. These results are Mean (SD).

a Other mood disorders includes obsessive-compulsive disorder, reaction to severe stress and adjustment disorders, dissociative and conversion disorders, soma-toform disorders, and other nonpsychotic mental disorders.

**Table 2:** Positive Airway Pressure (PAP) Usage in Years 1 and 2 in Unmatched Cohort.

	Adherent (A)	Intermediate (I)	Non-Adherent (N)	*p*-Value^†^
Year 1, Mean (SD)				
Hours per Day	7.1 (1.4)	3.4 (1.9)	0.5 (0.6)	<0.001
Hours per Use	7.3 (1.2) Day	5.7 (1.3)	3.2 (1.6)	<0.001
Days per Week	6.7 (0.4)	4.1 (1.9)	0.9 (1.0)	<0.001
Year 2, Mean (SD)				
Hours per Day	7.2 (1.5)	1.9 (2.2)	0.1 (0.3)	<0.001
Hours per Use	7.5 (1.3) Day	5.4 (1.8)	3.2 (1.9)	<0.001
Days per Week	6.7 (0.5)	2.3 (2.4)	0.2 (0.6)	<0.001

PAP adherent patients met US Centers for Medicare and Medicaid Services (CMS) criteria for eight of eight quarters over two years; intermediate patients met CMS criteria in at least one but no more than seven quarters; non-adherent patients did not meet CMS criteria for any of the eight quarters.

† Kruskal-Wallis rank sum test.

**Table 3:** Healthcare Resource Use and Costs in Years 1 and 2 in Inverse Probability Treatment Weighted Cohort by Positive Airway Pressure (PAP) Adherence.

	PAP Adherence				p-value^†^	
	Adherent (A)	Intermediate (I)	Non-Adherent (N)	A-I	A-N	I-N
Visits, Mean (SD)						
Year 1						
Office Visits	15.14 (14.13)	15.44 (14.16)	14.49 (13.70)	0.170	<0.001	<0.001
Specialist Visits	5.26 (12.43)	4.95 (11.56)	4.35 (10.05)	0.969	0.049	0.014
ER Visits	0.73 (1.88)	0.86 (2.04)	1.00 (2.42)	<0.001	<0.001	<0.001
All-Cause Hospitalizations	0.15 (0.67)	0.21 (0.78)	0.22 (0.76)	<0.001	<0.001	0.367
30-Day Readmissions	0.02 (0.40)	0.04 (0.47)	0.03 (0.36)	<0.001	<0.001	0.234
Depression Hospitalization	0.01 (0.21)	0.02 (0.28)	0.02 (0.18)	0.950	0.505	0.393
Self-Harm Events	0.04 (0.35)	0.06 (0.60)	0.05 (0.40)	<0.001	<0.001	0.146
Year 2						
Office Visits	12.96 (13.80)	12.58 (13.43)	12.09 (13.01)	0.001	<0.001	<0.001
Specialist Visits	4.49 (11.75)	3.99 (10.15)	3.60 (9.48)	0.016	<0.001	0.044
ER Visits	0.66 (1.92)	0.76 (1.86)	0.86 (2.18)	<0.001	<0.001	<0.001
All-Cause Hospitalizations	0.13 (0.54)	0.17 (0.72)	0.17 (0.70)	<0.001	<0.001	0.448
30-Day Readmissions	0.02 (0.24)	0.03 (0.41)	0.03 (0.38)	0.004	<0.001	0.050
Depression Hospitalization	0.01 (0.12)	0.02 (0.26)	0.01 (0.15)	0.003	0.019	0.765
Self-Harm Events	0.04 (0.43)	0.05 (0.40)	0.05 (0.34)	0.133	0.138	0.938
Costs, Mean (SD)						
Year 1						
Total	13,086 (18,560)	14,031 (20,098)	13,887 (19,239)	<0.001	0.969	<0.001
Inpatient Hospitalizations	1804 (7884)	2698 (10,818)	2804 (10,376)	<0.001	<0.001	0.389
Outpatient Hospitalizations	2238 (4680)	2399 (4815)	2293 (4610)	<0.001	0.910	<0.001
ER Visits	794 (2175)	984 (2610)	1118 (2996)	<0.001	<0.001	<0.001
Year 2						
Total	11,847 (18,909)	12,140 (19,426)	11,955 (20,083)	0.125	<0.001	0.006
Inpatient Hospitalizations	1634 (7890)	2240 (9980)	2274 (10,746)	<0.001	<0.001	0.412
Outpatient Hospitalizations	1986 (4400)	2134 (4871)	2001 (4622)	0.870	0.039	0.009
ER Visits	760 (2400)	896 (2439)	1006 (2714)	<0.001	<0.001	<0.001

PAP adherent patients met US Centers for Medicare and Medicaid Services (CMS) criteria for eight of eight quarters over two years; intermediate patients met CMS criteria in at least one but no more than seven quarters; non-adherent patients did not meet CMS criteria for any of the eight quarters.

† Wilcoxon rank-sum test for complex survey samples.

## Abbreviations:

CMS: Centers for Medicare and Medicaid Services
CPT: Current Procedural Terminology
HCPCS: Healthcare Common Procedure Coding System
HCRU: healthcare resource use
ICD: International Classification of Diseases
NNT: number needed to treat
OSA: obstructive sleep apnea
PAP: positive airway pressure
