# Supplementary material for: Depression and comorbid obstructive sleep apnea: Association between positive airway pressure adherence, occurrence of self-harm events, healthcare resource utilization, and costs
Source: J Affect Disord. Author manuscript; Available in PMC 2024 Sep 15. (PMC10922426; doi:10.1016/j.jad.2023.12.055)
Supplement: sup [file NIHMS1956043-supplement-sup.docx]

**SUPPLEMENT**

**Supplemental Table 1.** ICD-10 codes for conditions

| **Condition** | **ICD-10 Codes** |
| --- | --- |
| Anxiety | F40.x – F41.x |
| Asthma | J45.x |
| Atrial fibrillation | I48.0x-I48.2x, I48.91 |
| Atrial flutter | I48.3x-I48.4x, I48.92 |
| Bipolar disorder | F31.x |
| Cancer | Cxx.x |
| Central sleep apnea | G47.31 |
| Cerebrovascular disease | I6x.x |
| Chronic fatigue syndrome | R53.82 |
| COPD | J41.x-J44.x |
| Coronary artery disease | I20.x – I25.x |
| Depression | F32.x, F33.0, F33.1, F33.2, F33.3, F33.40, F33.41, F33.42, F33.9, F34.1, F39 |
| Dialysis use | Z99.2 |
| End-Stage Renal Disease | N18.6 |
| Fibromyalgia | M79.7 |
| GERD | K21.x |
| Heart failure | I11.0, I13.0, I50.x, I97.13x, I09.81 |
| Hyperlipidemia | E78.0x-E78.5x |
| Hypertension | I10, I11.x, I12.x, I13.x, I15.x |
| Insomnia | G47.0x, F51.0x |
| Morbid obesity | E66.01, E66.2, Z68.4x |
| Nocturnal hypoventilation | G47.36 |
| Obesity | E66.09, E66.1, E66.8, E66.9, Z68.3x |
| Obstructive sleep apnea | G47.33 |
| Other arrhythmia | I47.x, I49.x |
| Other mood disorders | F42.x, F43.x, F44.x, F45.x, F48.x |
| Overweight | E66.3, Z68.25-Z68.28 |
| Pneumonia | J12.x-J18.x |
| Pregnancy | Z32.01, Z33.x, Z36.x, Z37.x |
| Psychotic disorders | F20.x-F25.x, F28.x - F30.x |
| Pulmonary hypertension | I27.2x |
| Type 2 diabetes | E11.x |

**Supplemental Table 2.** HCPCS codes

| **Variable** | **HCPCS Codes** |
| --- | --- |
| Sleep test | 95808, 95810, 95811, G0398-G0400 |
| PAP equipment | A4604, A7027, A7028, A7029, A7030, A7031, A7032, A7033, A7034, A7035, A7036, A7037, A7038, A7039, A7044, A7046, E0464, E0466, E0470, E0471, E0561, E0562, E0601 |

**Supplemental Table 3.** CPT and ICD-10 codes for visits

| **Visit** | **Codes** |
| --- | --- |
| Office visits | **CPT:** 99201, 99202, 99203, 99205, 99211, 99212, 99213, 99214, 99215 |
| ER visits | **CPT:** 99281, 99282, 99283, 99284, 99285, 99288 |
| Specialist visits | **CPT:** 90791, 90792, 90832, 90833, 90834, 90836, 90837, 90839, 90840, 90845, 90846, 90847, 90849, 90853, 96150, 96151, 96152, 96153, 96154, 96155;  **ICD-10-PCS:** GZ10ZZZ, GZ11ZZZ, GZ12ZZZ, GZ13ZZZ, GZ14ZZZ, GZ2ZZZZ, GZ3ZZZZ, GZ50ZZZ, GZ51ZZZ, GZ52ZZZ, GZ53ZZZ, GZ54ZZZ, GZ55ZZZ, GZ56ZZZ, GZ58ZZZ, GZ59ZZZ, GZ60ZZZ, GZ61ZZZ, GZ63ZZZ, GZ72ZZZ, GZB0ZZZ, GZB1ZZZ, GZB2ZZZ, GZB3ZZZ, GZB4ZZZ, GZC9ZZZ, GZFZZZZ, GZGZZZZ, GZH, GZHZZZZ, GZJZZZZ |
| Self-Harm events | **ICD-10:** F.x and **ICD-10:** T36.x - T50.x, T52.x – T65.x, S51.x, S55.x, S59.x, S61.x, S65.x, S69.x;  Hospitalization or ER with **ICD-10:** T39.x, T42.x, T43.x, T58.x;  **ICD-10:** X8.x, X7.x |
